# Genital Irritation

**Published:** 1882-06

**Authors:** 


### GENITAL IRRITATION.

From a study of nineteen cases, Dr. Landon Carter Gray believes:

1. That there is no proof that genital irritation can produce a reflex paralysis.

2. That while it is probable that slight nervous disorders, as incontinence, retention, difficult micturition, erratic movements, and slight nervous disturbances, can be produced by genital irritation, the proof is not yet complete.

3. That operations for the removal of genital irritation may be beneficial even in organic nervous disease.

4. That we should, therefore, remove such genital irritation, if it exists in any case whatsoever, and thus give our patients the benefit of the doubt.

5. That in all cases of nervous disorders, with accompanying genital irritation, we should not regard the latter as the cause of the former until all other probable or even possible causes have been rigidly excluded.

6. That operations upon the genitals, even if there be no genital irritation, may prove to be a useful therapeutic measure in certain cases.—*Ann. of Anat. and Surg.*

